# Supplementary material for: IAF, QGF, and QDF Peptides Exhibit Cholesterol-Lowering Activity through a Statin-like HMG-CoA Reductase Regulation Mechanism: In Silico and In Vitro Approach
Source: Int J Mol Sci. 2021 Oct 14;22(20):11067. doi: 10.3390/ijms222011067 (PMC8538380; doi:10.3390/ijms222011067)
Supplement: Supplementary file 1 [file ijms-22-11067-s001.zip › ijms-1389885-supplementary.pdf]

Supplementary Material

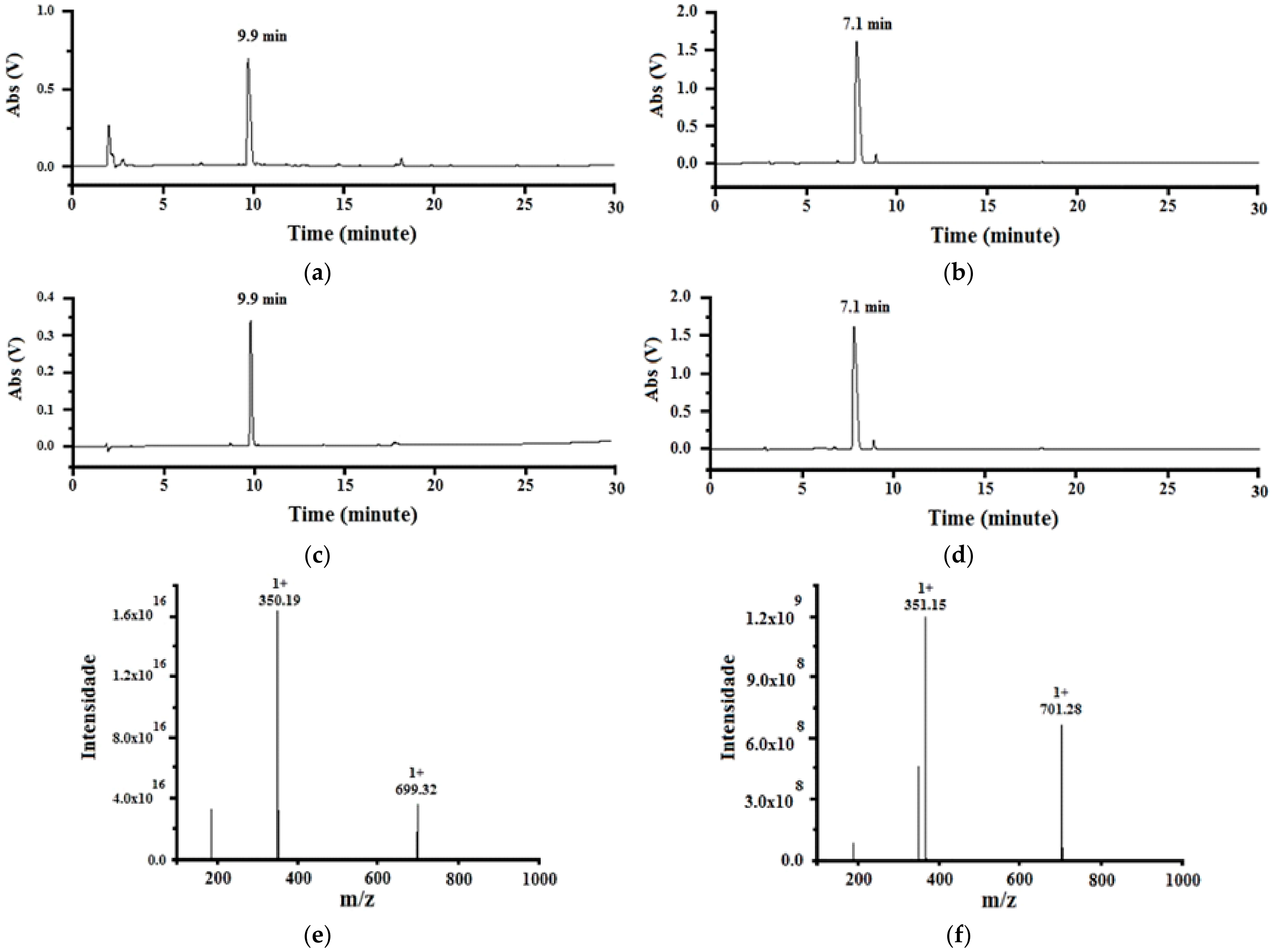

Figure S1. RP-HPLC chromatogram of the synthetic peptides. Chromatographic profile before purification (**a**,**b**); after the purification step (**c**,**d**); and mass spectrum (m/z, mass to charge ratio) of the IAF and QGF peptides (**e**,**f**), respectively.

Table S1. Experimental data of peptide synthesis.

Peptides	Synthesis Yield (%)	Purity (%)	$MW_{Theoretical}$ (g/mol)	$MW_{Measured}$ (g/mol)
QGF	78.9	96.7	350.4	351.2
IAF	74.6	98.1	349.4	350.2
QDF	68.2	95.0	408.4	408.2

MW: molecular weight.
